# Antidepressant-Like Effects of Low- and High-Molecular Weight FGF-2 on Chronic Unpredictable Mild Stress Mice

**DOI:** 10.3389/fnmol.2018.00377

**Published:** 2018-10-12

**Authors:** Lin Wang, Xi-Xi Li, Xi Chen, Xiao-Yan Qin, Elissavet Kardami, Yong Cheng

**Affiliations:** ^1^Key Laboratory of Ethnomedicine for Ministry of Education, Center on Translational Neuroscience, College of Life and Environmental Sciences, Minzu University of China, Beijing, China; ^2^School of Pharmacy, Minzu University of China, Beijing, China; ^3^Institute of Cardiovascular Sciences, St. Boniface Hospital Albrechtsen Research Centre, University of Manitoba, Winnipeg, MB, Canada

**Keywords:** HMW FGF-2, LMW FGF-2, depression, oxidative stress, AKT, ERK, Bcl-2, Caspase-3

## Abstract

The occurrence of depressive disorder has long been attributed to changes in monoamines, with the focus of drug treatment strategies being to change the effectiveness of monoamines. However, the success achieved by changing these processes is limited and further stimulates the exploration of alternative mechanisms and treatments. Fibroblast growth factor 2 (FGF-2), which occurs in a high-molecular weight (HMW) and low-molecular weight (LMW) form, is a potent developmental modulator and nervous system regulator that has been suggested to play an important role in various psychiatric disorders. In this study, we investigated the antidepressant effects of HMW and LMW FGF-2 on depression induced by chronic stress. Both peripheral LMW and HMW FGF-2 attenuated the depression-like behaviors in chronic unpredictable mild stress (CUMS) mice to a similar extent, as determined by the forced swimming, tail suspension, and sucrose preference tests. We then showed that CUMS-induced oxidative stresses in mice were inhibited by FGF-2 treatments both in central and peripheral. We also showed that both forms of FGF-2 increased the phosphorylation of ERK and AKT, increased Bcl-2 expression and inhibited caspase-3 activation in CUMS mice. Interestingly, HMW FGF-2 enhanced the activity of the brain-derived neurotrophic factor (BDNF) to a greater extent than did LMW FGF-2 in the hippocampus. Taken together, these results suggest that depressive symptoms can be relieved by administering different forms of FGF-2 peripherally in a CUMS-induced depression model through a similar antidepressant signaling pathway, therefore suggesting a potential clinical use for FGF-2 as a treatment for depression.

## Introduction

With rapid economic and societal development, stress and adversity have become serious physiological and psychological risks. Prolonged exposure is considered to be the main cause of many chronic diseases, especially depression (Kelly and Ismail, 2015), which is one of the most commonly diagnosed mental illnesses. Depression is projected to be the greatest cause of chronic disease in 2030, according the World Health Organization (World Health Organization [WHO], 2006). Furthermore, the pathogenesis of depression is complex and has often been linked to interactions among social, psychological, genetic, and neurobiochemical changes. Currently, the incidence of depression and the disability rate related to the disease are very high, and the lack of adequate treatment for this disease remains a matter of critical concern (Kessler et al., 2003). Due to low efficacy or intolerable side effects (Rush et al., 2006), nearly 50% of depressive patients do not respond to antidepressant treatment (Bschor et al., 2012). Another possible reason underlying treatment failure is our incomplete understanding of the etiology and pathophysiology of depression despite extensive research (Friedman, 2014). The monoamine hypothesis has historically dominated the pathophysiological view of depression (Chiu et al., 2017), and the most frequently prescribed medications for depression target increased extracellular serotonin, norepinephrine, and dopamine levels (Sargin et al., 2016; Belujon and Grace, 2017; Bighelli et al., 2018) by inhibiting monoamine reuptake by presynaptic terminals. Despite rapid changes in extracellular monoamine levels after drug administration, antidepressant effects develop slowly over several weeks of successive treatment (Nestler et al., 2002).

Duman and Monteggia (2006) initially proposed the neurotrophin factor hypothesis of depression, which is supported by studies demonstrating that a decrease in neurotrophic factors is the major element triggering depression (Fournier and Duman, 2012). Neurotrophic and other growth factors are likely to exert potent antidepressant effects, as they presumably increase neurogenesis, strengthen neuronal networks and potentiate regenerative responses to nerve insults (Malberg, 2004; Chen et al., 2010). As one of the most common neurotrophic factors, fibroblast growth factor 2 (FGF-2), also known as basic FGF, has attracted attention for its widespread activity, including roles in proliferation, migration, differentiation, and survival, as well as the induction of neurite outgrowth (Kasai et al., 2010). FGF-2 comprises at least four isoforms with diverse molecular weights, which are produced by the alternative translation of a single human mRNA transcript (Yu et al., 2008). Moreover, the 17/18 kDa protein, also called low-molecular weight (LMW) FGF-2, predominately resides in the cytoplasm, although a fraction of LMW FGF-2 is also localized to the nucleus (Chlebova et al., 2009). High-molecular weight (HMW) FGF-2 includes 22, 22.5, and 24 kDa forms are mostly reside in the nucleus and nucleolus. In addition, the different FGF-2 isoforms are found in the extracellular space, exerting paracrine and autocrine activities (Santiago et al., 2014). FGF-2 is known to be sensitive to stress exposure, and postmortem analyses of brains from patients with major depressive disorder (MDD) revealed significant reductions in FGF-2 mRNA levels in the hippocampus (Gaughran et al., 2006). FGF-2 and its receptors were downregulated in depressed subjects, and antidepressant treatments were shown to upregulate FGF-2 expression in humans and rodents (Evans et al., 2004; Bachis et al., 2008). In addition, the central administration of FGF-2 improved depression-like behaviors in several animal models of depression (Turner et al., 2008; Elsayed et al., 2012). However, most of these studies examined the role of central LMW FGF-2 in animal models of depression, which limits the potential of FGF-2 for depression in clinical use.

To further investigate the potential of FGF-2 as a therapeutic target for depression, we explored the antidepressant effects of peripheral FGF-2 on a chronic unpredictable mild stress (CUMS) model. We then assessed the influences of LMW and HMW FGF-2 on oxidative stress as a cellular index of antidepressant efficacy. The interdependency between the development of affective disorders and oxidative stress may be due to the frangibility of the central nervous system to oxidative damage (Balmus et al., 2016). We also detected the protein expression of brain-derived neurotrophic factor (BDNF) in CUMS mice, as numerous reports have underlined the fundamental role of BDNF in the pathology, physiology and treatment of depression (Sharma et al., 2016). For example, the BDNF levels were significantly reduced in the blood of patients with MDD (Karege et al., 2002) but were restored to baseline after antidepressant treatment (Lee and Kim, 2008). Furthermore, we demonstrate for the first time that different FGF-2 isoforms restored ERK and AKT signaling in the stressed mice.

## Materials and Methods

### Animal Preparation

Male C57BL/6 mice (4 weeks old, 25 ± 2 g) were purchased from Vital River (Beijing, China). The animals were raised at 23 ± 1°C and 50 ± 1% relative humidity under a 12-h light/dark cycle (lights on from 8 a.m. to 8 p.m.) and provided *ad libitum* access to a standard diet and drinking water. All animal experiments in this study were conducted in accordance with the National Institutes of Health Laboratory Animal Care and Use Guidelines (NIH Publication No. 80-23) and were approved by the Animal Care and Use Committee of Minzu University of China.

### Reagents

Assay kits for measuring superoxide dismutase (SOD), malondialdehyde (MDA), total glutathione/oxidized glutathione (T-GSH/GSSG), nitric oxide (NO), and total antioxidant capacity (T-AOC) were purchased from Nanjing Jiancheng Institute of Biotechnology (Nanjing, China). Anti-phospho-FGFR1 (Tyr653+Tyr654) polyclonal antibody (rabbit) was purchased from Bioss (Beijing, China). BDNF polyclonal antibody (rabbit) was purchased from Cloud-Clone Corp (Wuhan, Beijing). Goat-anti mouse IgG, goat-anti rabbit IgG, β-actin monoclonal antibody (rabbit), AKT (pan) monoclonal antibody (mouse), phospho-AKT (Ser473) monoclonal antibody (rabbit), phospho-p44/42 MAPK (Erk1/2) (Thr202/Tyr204) monoclonal antibody (rabbit), Erk1/2 monoclonal antibody (mouse), Bcl-2 monoclonal antibody (rabbit), and caspase-3 polyclonal antibody (rabbit) were purchased from Cell Signaling Technology (Danvers, United States). FGF-2 was produced in the EK’s lab, and the purification data, western blot data and activity data were presented in **Supplementary Figure 1**.

### Experimental Groups

All animals were housed for 1 week to adapt to the environment, and were then randomly assigned to one of four independent groups: control group, CUMS+saline (NS) group, CUMS+HMW FGF-2 (5 ng/g) group, and CUMS+LMW FGF-2 (5 ng/g) group. The number of animals used to measure each parameter in each group is shown in **Table 1**.

**Table 1:** The number of mice in different groups and various testing experiments.

	Control	CUMS+NS	CUMS+HMW FGF-2 (5 ng/g)	CUMS+LMW FGF-2 (5 ng/g)	Total
Behavioral test	(15)	(12)	(12)	(12)	(51)
Oxidative stress in tissue and serum	(6)	(6)	(6)	(6)	(24)
Western blot	(5)	(5)	(5)	(5)	(20)
Total	15	12	12	12	51


*The numbers in parentheses represent the number of animals used in the corresponding experiment.*

### Drug Administration

The C57BL/6 mice were subjected to CUMS without drug treatment for 45 days. The LMW (18 kDa) and HMW FGF-2 used in the procedures were dissolved in normal saline, and both were then injected at a dose of 5 ng/g intraperitoneally (i.p.) according to the methods of a previous study (Perez et al., 2009).

### Experimental Design and CUMS Program

**FIGURE 1:**
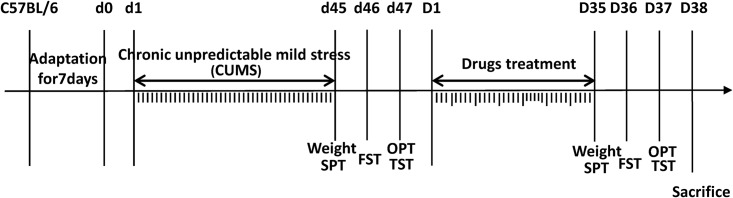
Schematic diagram of the experimental procedure, as described in the Section “Materials and Methods.” The behavioral tests, including the sucrose preference test, forced swimming test, open-field test, and tail suspension test, were performed after chronic unpredictable mild stress (CUMS) and before sacrifice. Mice were subjected to different kinds of mild stressors for 45 days preceding drug treatment to induce CUMS. LMW and HMW FGF-2 treatment were administered every other day after CUMS and continued for 5 weeks. All mice were sacrificed following the behavioral tests. d1–d45 indicates the days animals subjected to CUMS; D1–D35 indicates the days animals received the FGF-2 treatments.

The illustration in **Figure 1** outlines the experimental procedure that we established in this study. Before i.p. injection and after drug treatment, the sucrose preference test (SPT), forced swimming test (FST), open-field test (OFT) and tail suspension test (TST) were performed, and body weights were measured before each behavioral test. The CUMS model applied in our study includes 10 sources of stress: (1) 5 min tail suspension, (2) 5 min cold swim at 4–6°C, (3) clipping the distal 1 cm of the tail tip with tongs for 5 min, (4) 5 min 45°C hot water forced swimming, (5) 24 h food fasting, (6) 24 h water fasting, (7) 24 h of damp padding (200 mL of water per 100 g of padding), (8) 24 h cage tilting (cage inclined at 45° with respect to the horizontal), (9) 24 h of day and night reversal, and (10) 6 h of constraint. Mice in the CUMS+NS, CUMS+HMW FGF-2 (5 ng/g), and CUMS+LMW FGF-2 (5 ng/g) groups were exposed to CUMS for 45 days, and two randomly selected pressure sources were applied to the animals daily. At the same time, animals in the control group maintained a normal feeding schedule. HMW or LMW FGF-2 was administered once every 2 days starting on the sixth week and continued for consecutive 5 weeks, while animals in the CUMS+NS group were given the same volume of normal saline on the same schedule. Then, each group of mice was sacrificed after the end of behavior tests.

### Behavioral Tests

To remove olfactory disturbances, trial chambers were wiped with 75% alcohol in two separate TST and OFT sessions, while the water used in the FST was changed frequently between each mouse trial. In addition, the animals were blinded to the experimenters for the behavioral tests.

### Sucrose Preference Test

The SPT, which can characterize a lack of pleasure, one of the primary symptoms of human depression, has been extensively used to assess depressive behavior in animals. Based on the collected data, the SPT was implemented for three consecutive days (Yang Y. et al., 2017). During the first 24 h, mice were given free access to two bottles of 1% sucrose solution to acclimate them to the sucrose solution. On the second day, one bottle of sucrose solution was replaced with the same volume of drinking water. On the third day, the mice were deprived of food and water for 20 h and were then formally subjected to the SPT. During the experiment, each mouse was offered two identical bottles, one containing a 1% sucrose solution and the other containing pure drinking water, and fluid consumption was evaluated after a 4 h test. Sucrose preference was calculated according to the following formula: sucrose preference proportion = sucrose solution consumption/total liquid consumption ^∗^ 100%.

### Forced Swimming Test

Mice subjected to the FST were placed in a glass cylinder (diameter, 10 cm, Tai League Software, Chengdu, China) filled with 14 cm of water (25 ± 1°C) for a period of 6 min; the immobility times of the mice during the final 5 min were recorded and analyzed using FST software. When mice gave up trying to escape and participated only in keeping their heads above water, they were considered immobile, denoting “behavioral despair.” After the experiment, the mice were simply dried and placed back into their housing cages.

### Open-Field Test

The OFT is used to evaluate the state of autonomic movement, aiming to identify agitation, and pathological behavior. Before the formal experiment, each mouse was carefully placed in the center of the trial chamber on (62.5 cm × 74 cm × 51 cm, Tai League Software, Chengdu, China). The movement of each moue was recorded for 5 min by a video camera, and the data were analyzed using OFT software.

### Tail Suspension Test

The TST is a classic behavioral test used to characterize the degree of desperation and helplessness in animal behavior. Before the test, the tail of the mouse was fixed on a bracket with adhesive tape, and the mouse was suspended upside down in the TST-100 tail suspension chamber (Tai-Co software, Chengdu, China). After 1 min of adaptation, TST software was used to record and analyze the immobility time of the mice over the subsequent 5 min (activity threshold value = 30%).

### Sacrifice and Sample Preparation

On the second day after the behavioral tests were completed, blood was collected and centrifuged at 3,500 rpm for 10–15 min at 4°C after incubation for 30 min at room temperature. Then, the serum was immediately aspirated, transferred to a fresh Eppendorf tube and stored at -80°C for further analysis. Following blood collection, the mice were sacrificed, and the prefrontal cortex and hippocampus were dissected from the brain tissue, quickly frozen in liquid nitrogen, and stored at -80°C until use.

### Oxidative Markers

Samples (0.5 g) of prefrontal cortex and hippocampal tissues were added to 300 μL of tissue lysis solution for protein extraction and incubated for 30 min on ice. The homogenates were centrifuged at 4°C for 15 min at 13,000 rpm, and the supernatants were transferred to Eppendorf tubes. The total protein content in the tissue lysates was subsequently measured using the BCA protein assay kit (Solarbio, Beijing, China). Both the tissue supernatants and serum samples were then used to investigate oxidation metabolic indicators. Oxidative status and antioxidant activity were quantified using the SOD (A001-3), MDA (A003-1), T-GSH/GSSG (A061-1), T-AOC (A015-1), and NO (A012-1) assay kits. All indicators were analyzed according to the manufacturers’ specifications.

### Western Blotting

Equal amounts of total protein (20 μg/lane) were separated by 10% SDS-PAGE (50–100 kDa) and electrotransferred to PVDF membranes. After blocking the membranes with TBST containing 5% non-fat dried milk for 1 h at room temperature, the membranes were incubated overnight with primary antibody at 4°C. Then, the membranes were washed three times with TBST for 10 min each and then incubated for 1 h with second antibody. After further washing, the protein bands were visualized using a chemiluminescence detection system (Tanon 4200, Shanghai, China). The immunoblot signals were analyzed using ImageJ software.

### Statistical Analysis

The data are presented as the mean ± SEM (standard error of the mean) using SPSS19.0 statistical analysis software. The data were analyzed using *t*-test or one-way ANOVA followed Tukey’s *post hoc* test. ^∗^*p* < 0.05, ^∗∗^*p* < 0.01, and ^∗∗∗^*p* < 0.001 were considered statistically significant.

## Results

### Depression Induced by Chronic Unpredictable Mild Stress (CUMS) in Mice

**FIGURE 2:**
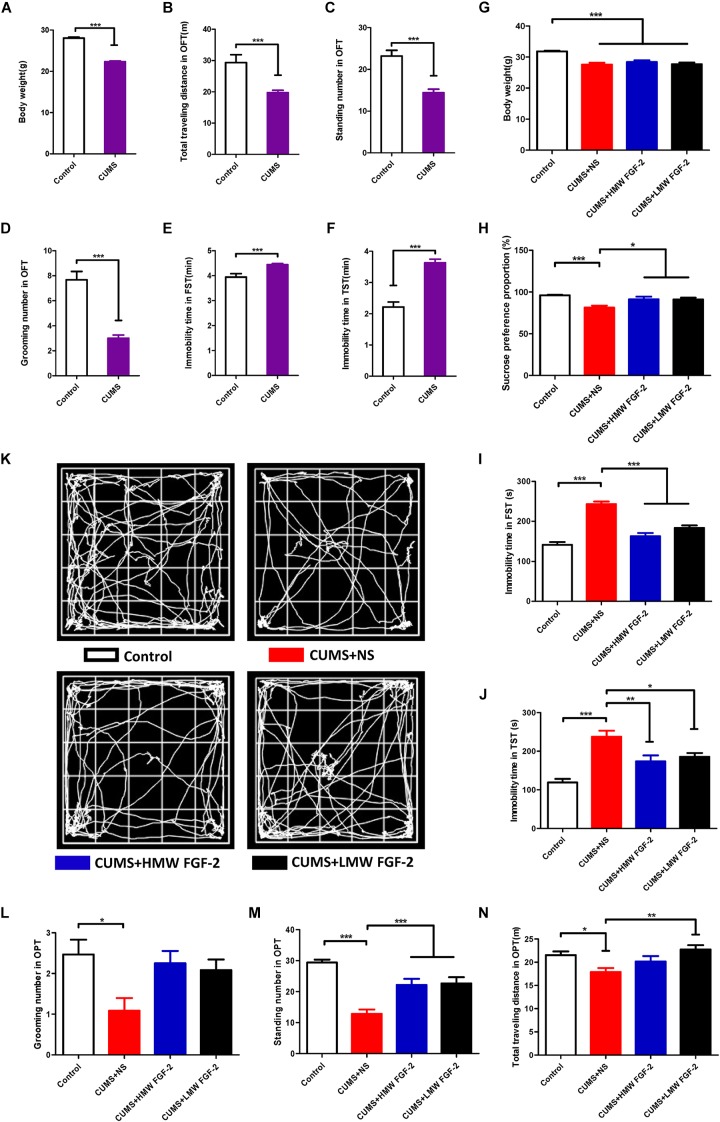
Chronic unpredictable mild stress (CUMS) leads to depression-like behaviors in mice, and FGF-2 reverses these behaviors. **(A–F)** Behavioral tests showing that performing CUMS procedures for 45 days can lead to depression. **(G)** All stressed mice showed markedly lower body weight treated with or without FGF-2. **(H)** Mice in the CUMS+HMW FGF-2 and CUMS+LMW FGF-2 groups displayed increased sucrose preference. **(I)** Mice in the CUMS+HMW FGF-2 and CUMS+LMW FGF-2 groups showed significantly increased forced swimming immobility time and **(J)** significantly increased tail suspension immobility time. **(K)** Trace plot of the open-field test after FGF-2 administration. **(L)** Open-field test showing the pathological behavior of animals. No significant differences in the grooming numbers were observed in the drug treatment groups. **(M)** The standing numbers were significantly increased by HMW FGF-2 and LMW FGF-2 treatment, **(N)** the total distance traveled by CUMS mice was significantly increased by treatment with LMW FGF-2. Results are expressed as the mean ± SEM, **(A–F)**, *t*-tests; **(G–J, L–N)**, one-way ANOVA with Tukey’s *post hoc* test, *n* = 15, 12, ^∗^*p* < 0.05, ^∗∗^*p* < 0.01, and ^∗∗∗^*p* < 0.001.

To assess the impact of the CUMS procedure, the mice mood states were evaluated by the OPT, FST, and TST as well as by their body weights. If CUMS mice showed significant changes in all of these tests, they were deemed CUMS-induced depression mice. As shown in **Figures 2A–F**, the body weights of the CUMS mice (**Figure 2A**, *n* = 36) and control mice (**Figure 2A**, *n* = 15) were 22.34 ± 0.21 g and 28.06 ± 0.23 g, respectively. The weights of the CUMS mice versus those of the control mice were decreased significantly (**Figure 2A**, *p* < 0.001). In the OPT, the total distances traveled were 19.72 ± 0.76 m in CUMS mice (**Figure 2B**, *n* = 36) and 29.33 ± 2.54 m in control mice (**Figure 2B**, *n* = 15, *p* < 0.001); the standing numbers were 14.42 ± 0.83 in CUMS-induced depression mice (**Figure 2C**, *n* = 36) and 23.20 ± 1.36 in control mice (**Figure 2C**, *n* = 15, *p* < 0.001), while the grooming numbers were 3.00 ± 0.26 in CUMS mice (**Figure 2D**, *n* = 36) and 7.67 ± 0.67 in control mice (**Figure 2D**, *n* = 15, *p* < 0.001). The immobility times in the FST were 4.44 ± 0.05 min in CUMS-induced depression mice (**Figure 2E**, *n* = 36) and 3.94 ± 0.14 min in control mice (**Figure 2E**, *n* = 15, *p* < 0.001), and the immobility times in the TST were 3.63 ± 0.12 min in CUMS mice (**Figure 2F**, *n* = 36) and 2.21 ± 0.16 min in controls (**Figure 2F**, *n* = 15, *p* < 0.001). Thus, CUMS leads to depression-like behavior in mice.

### FGF-2 Reverses the Depression-Like Behavior Induced by CUMS

Body-weight measurement is typically used as a non-specific indicator of poor health in rodents, and we thus measured the mice body weights after 5 weeks of drug administration. The average body weights of the mice were 31.83 ± 0.19 g in the control group, 27.57 ± 0.60 g in the CUMS+NS group, 28.38 ± 0.58 g in the CUMS+HMW FGF-2 group, and 27.69 ± 0.53 g in the CUMS+LMW FGF-2 group. As shown in **Figure 2G**, the body weights among the three CUMS groups (CUMS+NS, CUMS+HMW FGF-2, and CUMS+LMW FGF-2 group) were lower than those in the control group, and statistical analysis indicated no significant differences in the mouse body weights after drug treatment (*p* > 0.05), suggesting that FGF-2 cannot ameliorate CUMS-induced reductions in body weight.

To confirm the underlying antidepressant function of FGF-2, behavioral analyses were carried out. Compared with control mice, the depressed mice showed lower sucrose preference (**Figure 2H**, *p* < 0.001) and longer immobility times in both the FST (**Figure 2I**, *p* < 0.001) and TST (**Figure 2J**, *p* < 0.001). However, the sucrose preference levels in both the CUMS+HMW FGF-2 (**Figure 2H**, *p* < 0.05) and CUMS+LMW FGF-2 (**Figure 2H**, *p* < 0.05) groups were higher than those in the CUMS+NS group. For the FST, the CUMS+HMW FGF-2 (**Figure 2I**, *p* < 0.001) and CUMS+LMW FGF-2 (**Figure 2I**, *p* < 0.001) groups exhibited shorter immobility times than the CUMS mice. Similarly, compared with the CUMS+NS group, the immobility time in the TST was shorter in the CUMS+HMW FGF-2 (**Figure 2J**, *p* < 0.01) and CUMS+LMW FGF-2 (**Figure 2J**, *p* < 0.05) groups. Mice were naturally fond of approaching a protective wall instead of being exposed to outdoor danger, but the instinct for competitive foraging prompted them to explore (Christakis et al., 2012). Depressed mice spent significantly more time resting in the middle and even entered the center significantly more often, and they also traveled less than control mice did (**Figure 2K**), suggesting that CUMS-induced depression mice were non-active. Meanwhile, the grooming numbers were not significantly different from those of the CUMS mice in the OFT after drug administration (**Figure 2L**, *p* > 0.05), whereas the standing numbers showed significant differences that were similar to those in the FST (**Figure 2M**, *p* < 0.001). The CUMS+NS group traveled a shorter total distance than the control group (**Figure 2N**, *p* < 0.05), but the CUMS+LMW FGF-2 mice traveled further than the CUMS mice after drug administration (**Figure 2N**, *p* < 0.01). In general, treatment with HMW or LMW FGF-2 injection ameliorated the depression-like behaviors.

### FGF-2 Alleviates Oxidative Stress in the Prefrontal Cortex and Hippocampus of CUMS-Induced Mice

**FIGURE 3:**
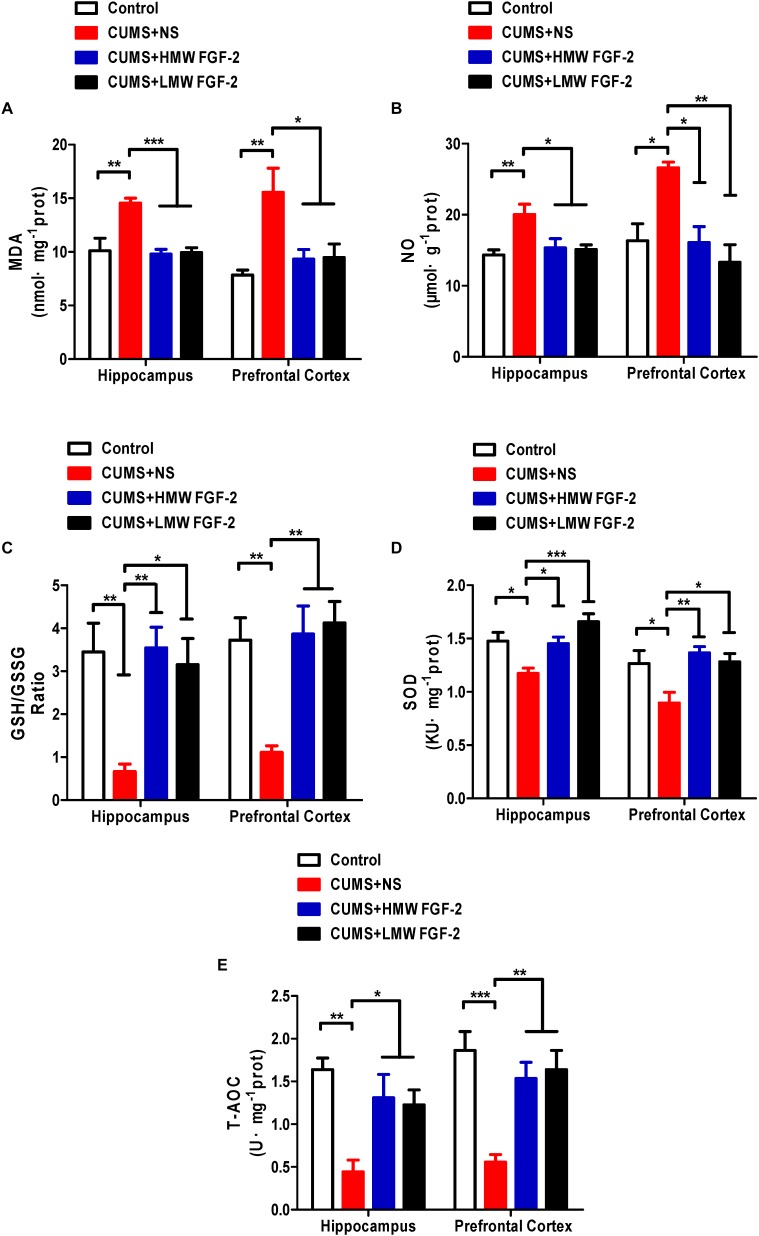
Effects of FGF-2 on the changes in five oxidative stress indices, MDA, NO, GSH, SOD and T-AOC, in the hippocampus and prefrontal cortex of CUMS-induced mice. **(A)** MDA content. **(B)** NO content. **(C)** GSH/GSSG ratio. **(D)** SOD activity. **(E)** T-AOC activity. Data are expressed as the mean ± SEM. Compared with those in the control group, MDA and NO levels were significantly increased, while SOD and T-AOC activity, as well as the GSH/GSSG ratio, were significantly decreased in the hippocampus and prefrontal cortex of CUMS mice. Furthermore, compared with those in the CUMS+NS group, MDA and NO levels were significantly decreased, while SOD and T-AOC activity, as well as the GSH/GSSG ratio, were significantly increased in the FGF-2-treated groups. **(A–E)**, one-way ANOVA with Tukey’s *post hoc* test, *n* = 6, ^∗^*p* < 0.05, ^∗∗^*p* < 0.01, and ^∗∗∗^*p* < 0.001.

To determine whether the antidepressant influence of FGF-2 involves oxidative stress, we first detected five oxidative stress indices in the hippocampus and prefrontal cortex. MDA is a marker of lipid peroxidation indicating oxidative stress levels, and excessive NO accumulation in vivo can also cause oxidative damage. Significant increases in the levels of MDA (**Figure 3A**, p < 0.01) and NO (**Figure 3B**, p < 0.01; p < 0.05) were observed in the hippocampus and prefrontal cortex of depressed mice compared with those in control mice. Moreover, MDA (**Figure 3A**, p < 0.001; p < 0.05) and NO (**Figure 3B**, p < 0.05; p < 0.01) levels in the hippocampus and prefrontal cortex were significantly reduced by FGF-2 treatment. These data indicated that FGF-2 improves CUMS-induced hippocampal and prefrontal cortex oxidative stress levels.

Furthermore, we investigated the GSH level and SOD and T-AOC activity in the same brain tissue of CUMS mice. The GSH/GSSG ratio (**Figure 3C**, p < 0.01) and the activity of SOD (**Figure 3D**, p < 0.05) and T-AOC (**Figure 3E**, p < 0.01; p < 0.001) were significantly decreased in the hippocampus and prefrontal cortex. However, treatment with FGF-2 significantly increased GSH levels, as well as SOD and T-AOC activity (**Figures 3C–E**), further indicating the protective effect of FGF-2 against CUMS-induced hippocampus and prefrontal cortex oxidative stress.

### FGF-2 Alleviates Oxidative Stress in the Serum of CUMS-Induced Mice

**FIGURE 4:**
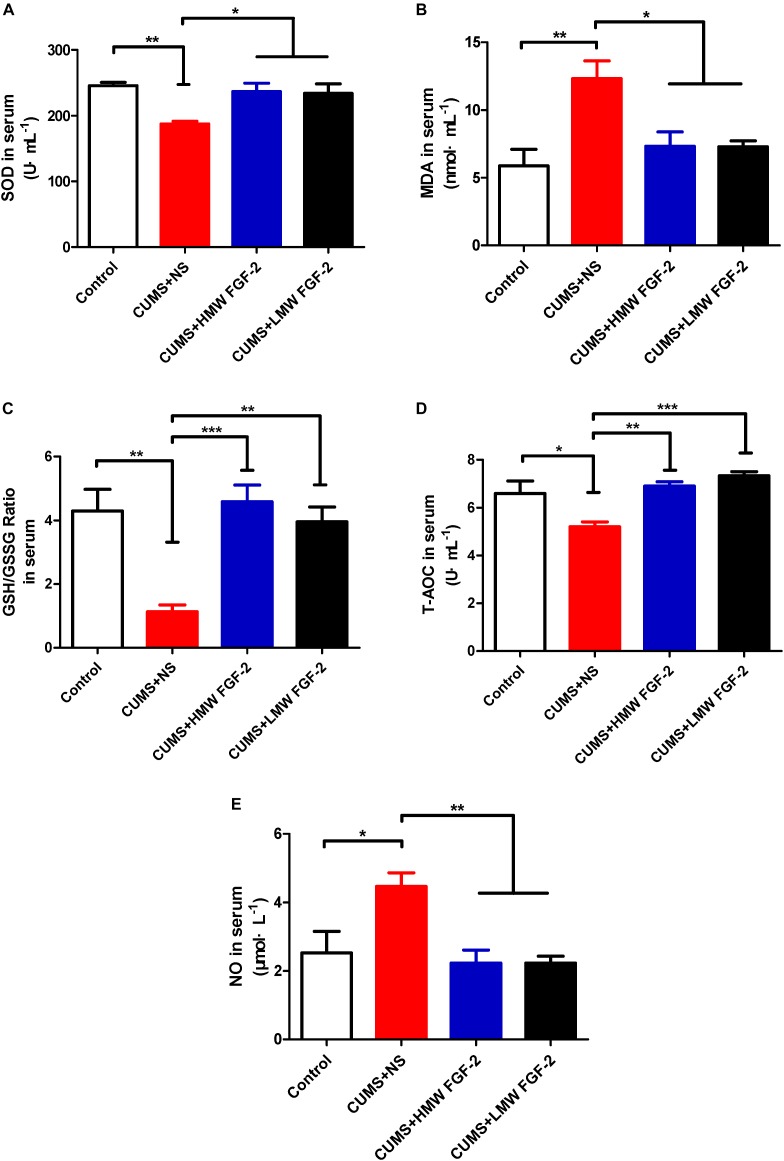
Effects of FGF-2 on the changes in SOD and T-AOC activity and MDA, GSH, and NO levels in CUMS mice serum. **(A)** SOD activity. **(B)** MDA content. **(C)** GSH/GSSG ratio. **(D)** T-AOC activity. **(E)** NO content. Data are expressed as the mean ± SEM. SOD and T-AOC activity, as well as GSH levels were significantly decreased, while MDA and NO levels were significantly increased in the CUMS+NS group compared to those in the control group. However, after HMW FGF-2 or LMW FGF-2 administration, SOD and T-AOC activity, as well as GSH levels were significantly increased, while MDA and NO levels were significantly decreased. **(A–E)**, one-way ANOVA with Tukey’s *post hoc* test, *n* = 6, ^∗^*p* < 0.05, ^∗∗^*p* < 0.01, and ^∗∗∗^*p* < 0.001.

To further confirm that FGF-2 improved depressive symptoms stemming from oxidative stress damage, we also investigated the influences of FGF-2 on the same oxidative stress indices, SOD, T-AOC, MDA, GSH, and NO, in serum. A significant reduction in the SOD antioxidant enzyme activity and a remarkable increase in MDA levels were observed in the CUMS+NS group (**Figures 4A,B**, p < 0.01; p < 0.01). The GSH/GSSG ratio and T-AOC activity were also significantly decreased after the CUMS procedure (**Figures 4C,D**, p < 0.01; p < 0.05). Moreover, NO production increased significantly after the CUMS procedure (**Figure 4E**, p < 0.05). Compared with the CUMS+NS group, MDA levels (**Figure 4B**, p < 0.05) and NO production (**Figure 4E**, p < 0.01) were significantly decreased by FGF-2 treatment, while the GSH/GSSG ratio (**Figure 4C**, p < 0.001; p < 0.01) and T-AOC activity (**Figure 4D**, p < 0.01; p < 0.001) were both significantly increased. Furthermore, two types of FGF-2 isoforms appeared to significantly enhance SOD activity (**Figure 4A**, p < 0.05). Both HMW and LMW FGF-2 treatment markedly improved all the antioxidant and oxidant indices in CUMS mice, especially the GSH/GSSG ratio and T-AOC activity, which were equal to or higher than those in the control group (**Figures 4C,D**).

### FGF-2 Activates FGFR1 and Upregulates BDNF Protein Expression in CUMS-Induced Mice

**FIGURE 5:**
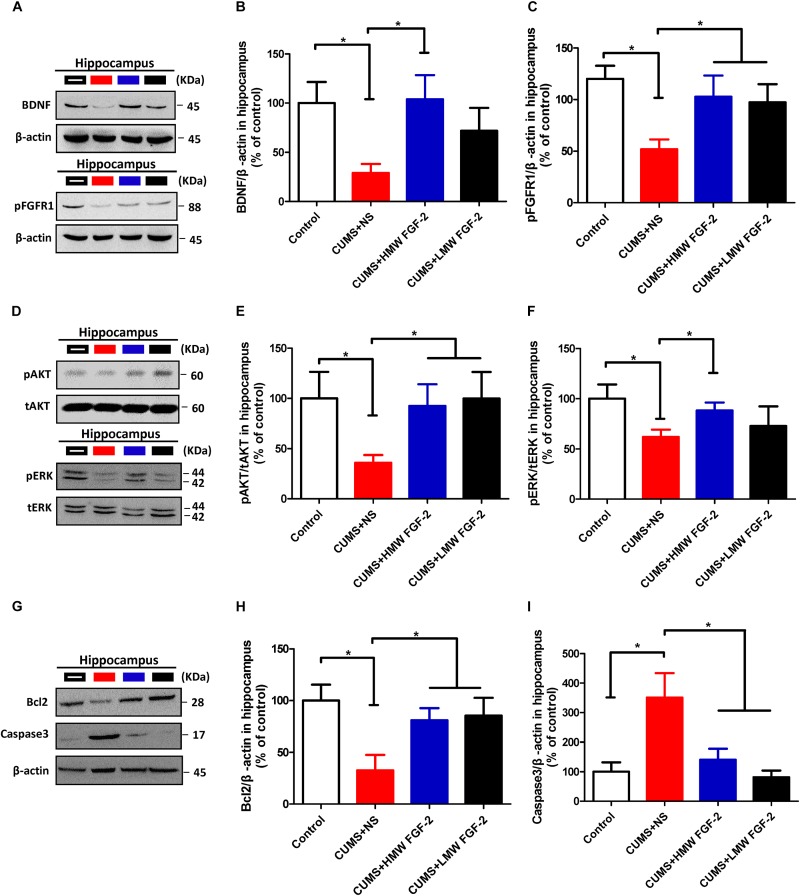
FGF-2 upregulates BDNF protein expression and activates the ERK and AKT signaling pathways by acting on FGFR1 in the hippocampus of CUMS-induced mice. Representative immunoblots of BDNF and pFGFR1 **(A)**, p-AKT/t-AKT and p-ERK/t-ERK **(D)**, and Bcl-2 and caspase-3 **(G)** detected by Western blotting in hippocampal tissues. The remaining panels depict the quantification of immunoblotting bands of BDNF **(B)**, p-FGFR1 **(C)**, p-AKT **(E)**, p-ERK **(F)**, Bcl-2 **(H)** and caspase-3 **(I)**. Panels **(B,C,E,F,H,I)** were analyzed by one-way ANOVA with Tukey’s *post hoc* test, *n* = 5, ^∗^*p* < 0.05, ^∗∗^*p* < 0.01, and ^∗∗∗^*p* < 0.001.

**FIGURE 6:**
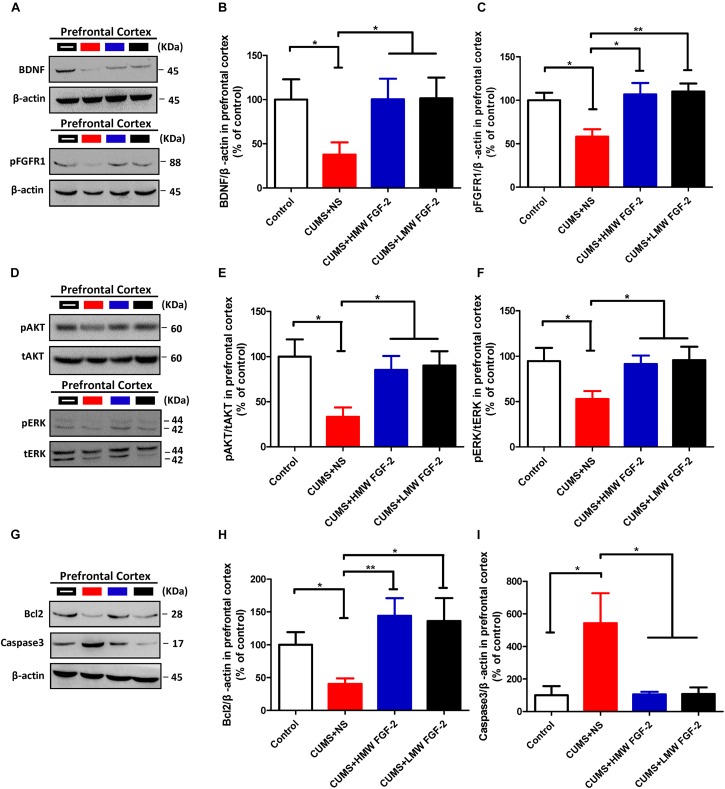
FGF-2 upregulates BDNF protein expression and activates the ERK and AKT signaling pathways by acting on FGFR1 in the prefrontal cortices of CUMS-induced mice. Representative immunoblots of BDNF and pFGFR1 **(A)**, p-AKT/t-AKT and p-ERK/t-ERK **(D)**, and Bcl-2 and caspase-3 **(G)** detected by Western blotting in tissues from the prefrontal cortex. The remaining panels depict the quantification of immunoblotting bands of BDNF **(B)**, p-FGFR1 **(C)**, p-AKT **(E)**, p-ERK **(F)**, Bcl-2 **(H),** and caspase-3 **(I)**. Panels **(B,C,E,F,H,I)** were analyzed by one-way ANOVA with Tukey’s *post hoc* test, *n* = 5, ^∗^*p* < 0.05, ^∗∗^*p* < 0.01, and ^∗∗∗^*p* < 0.001.

After 45 days of exposure to CUMS, BDNF protein expression and FGFR1 phosphorylation were significantly decreased in the hippocampus (**Figures 5A–C**, *n* = 5, *p* < 0.05). However, both markers were significantly increased and even nearly recovered to control levels after HMW FGF-2 treatment, but BDNF protein expression in the LMW FGF-2 group was not significantly different from that in the CUMS+NS group (**Figure 5B**, *n* = 5, *p* > 0.05). Furthermore, BDNF protein expression and FGFR1 phosphorylation in the prefrontal cortex were similar to those in the hippocampus (**Figures 6A–C**, *n* = 5, *p* < 0.05).

### Involvement of the AKT and ERK Pathways in FGF-2-Induced Antidepressant Effects in CUMS-Induced Mice

To determine whether AKT and ERK activation are involved in the effect of FGF-2 on depression, we first determined the levels of p-AKT and p-ERK in the hippocampus. Western blotting showed that treating CUMS mice with different molecular weights of FGF-2 significantly increased AKT phosphorylation compared with that in the CUMS+NS group (**Figures 5D,E**, *n* = 5, *p* < 0.05). A similar trend of increased ERK phosphorylation was observed, but only the levels in the HMW FGF-2 group were significantly different from those in the CUMS+NS group (**Figures 5D,F**, *n* = 5, *p* < 0.05).

To confirm that the improvement in the depressive symptoms of CUMS mice was associated with AKT and ERK activation, we further detected the same key proteins in the prefrontal cortex. Western blotting indicated that p-AKT was reduced in depressed mice (**Figures 6D,E**, *n* = 5, *p* < 0.05), and this tendency was reversed by treatment with different molecular weights of FGF-2 (**Figure 6E**, *n* = 5, *p* < 0.05). Moreover, similar to p-AKT, p-ERK (**Figure 6F**, *n* = 5, *p* < 0.05) levels were also significantly increased by FGF-2 treatment, indicating that the AKT and ERK pathways might be involved in the antidepressant effects of FGF-2.

### Antidepressant Effects of FGF-2 Are Related to Increased Bcl-2 Expression and Caspase-3 Inhibition

To further examine the antidepressant mechanism of FGF-2, we assessed the expression of AKT and ERK downstream targets, namely, the antiapoptotic protein Bcl-2 and the proapoptotic protein caspase-3, in the hippocampus and prefrontal cortex. Western blotting showed that CUMS significantly reduced Bcl-2 expression in the hippocampus compared with that in the controls (**Figures 5G,H**, *n* = 5, *p* < 0.05); however, treatment with both molecular weights of FGF-2 increased Bcl-2 expression compared with that in the CUMS+NS group (**Figure 5H**, *n* = 5, *p* < 0.05). In addition, active caspase-3 levels were significantly increased by CUMS compared with those in control mice (**Figure 5I**, *n* = 5, *p* < 0.05), and both FGF-2 isoforms significantly reversed the CUMS-induced changes in caspase-3 levels (**Figure 5I**, *n* = 5, *p* < 0.05). Moreover, the levels of Bcl-2 and active caspase-3 in the prefrontal cortex of CUMS mice showed a trend similar to that in the hippocampus after FGF-2 treatment (**Figures 6G–I**). These results suggested that FGF-2 may inhibit cell apoptosis by upregulating Bcl-2 protein expression and inhibiting caspase-3 activation.

## Discussion

Fibroblast growth factor 2 is a multipotent protein with various isoforms, which exhibit diverse biological functions. All FGF-2 isoforms originate from the alternative translation of a single mRNA: the 18 kDa form, known as LMW FGF-2, is initiated from the AUG codon in both mice and humans, while HMW FGF-2 translation initiates from the CUG codon upstream of the AUG, yielding two mouse (21–22 kDa) and four human (21–34 kDa) isomers (Liao et al., 2010a,b). While the biological functions of LMW FGF-2 have been thoroughly investigated, HMW FGF-2 is not well studied. Although the HMW FGF-2 is reported to localize to the nucleus predominately (Kole et al., 2017), it can also be found in the extracellular space under certain conditions, and the release of HMW FGF-2 into extracellular space is thought to go through vesicle shedding (Taverna et al., 2003; Boris et al., 2010). Some studies have emphasized the different functions of different FGF-2 isoforms. Timmer et al. (2004) showed that when Schwann cells overexpressing 18 kDa or 21/23 kDa FGF-2 co-transplanted with dopamine grafts into the brain of 6-hydroxydopamine-lesioned rats, 21/23 kDa FGF-2 had a better effect to enhance the survival and innervation of intrastriatal dopamine graftset al., 2. Another study showed that integrin pattern changes and cell migration were specific to LMW FGF-2 (Klein et al., 1996), whereas the HMW form restricted cell migration (Ding et al., 2002) and induced cell transformation or growth arrest depending on the cell type and expression level (Yu et al., 2007). In our study, both isoforms of FGF-2 had antidepressant effects on the CUMS depression mouse model, which was consistent with a previous report in which chronic treatment with exogenous FGF-2 decreased the anxious behavior in hereditary rats (Perez et al., 2009). This report investigated whether the impact of exogenous FGF-2 on anxiety originates from genetic predisposition, but environmental factors such as long-term and high-intensity stress can substitute for genetic vulnerability, particularly in individuals who are inclined to highly anxious behavior or even MDD. Thus, our experiments investigated exogenous peripheral FGF-2 in a CUMS mouse model of depression induced by environmental stimulation. The observed antidepressant effect of peripheral FGF-2 most likely to be a direct consequence of FGF-2 acting in the brain as FGF-2 has been demonstrated to cross the blood–brain barrier (Wagner et al., 1999; Jin et al., 2005), and also because of our results showing the peripheral FGF-2-induced activation of FGFR1 in the prefrontal cortex and hippocampus of CUMS-induced depression mice. However, we can’t exclude the possibility that pERK and pAKT may also be activated indirectly by peripheral FGF-2 in the animals.

Long-term pressure is a well-known risk factor for depression in humans with genetic vulnerability (Kendler et al., 2006). Investigators have developed animal models of stress (e.g., immobilization, unpredictable chronic stress, social isolation, and maternal deprivation) to determine the causal relationship between mechanism and disease (Phillips, 2017). One study described a model of CUMS depression in male rats to imitate unforecasted life stress sources that can lead to the development of MDD in humans (Sun et al., 2016). In this study, mice showed significant pleasure deficiency and behavioral hopelessness, which are typical manifestations of depression. The decreased sucrose preference in the SPT, as well as the significant increase in immobility time in the FST and TST, indicated the successful establishment of a depression model in mice. Decreased sucrose consumption in the SPT indicates inhibition of the brain reward system, which is a representative symptom of depression in rodents (Tatyana et al., 2004). Immobility times in the FST and TST indicate hopeless behavior, which is another notable symptom of depression (Han et al., 2015). While the OFT is often used to assess anxiety behaviors, it can also be used to evaluate spontaneous activity in rodents (Yang X.H. et al., 2017). In the present study, differences in OFT performance, including the total distance traveled and the times spent standing and grooming, were observed among the CUMS mice, indicating that CUMS significantly impacted exercise, exploration and anxiety levels in the mice. All these results showed that CUMS produced symptoms similar to depression-like symptoms in humans, including anhedonia, increased hopelessness, and behavioral and cognitive dysfunction, which was consistent with previous results (Yazir et al., 2012).

Many diseases are associated with oxidative stress injury, especially neurodegenerative diseases such as Alzheimer’s disease, Parkinson’s disease and schizophrenia, which are caused by the excessive production of oxygen free radicals and imbalance of antioxidant capacity (Vaváková et al., 2015). Mitochondrial oxidative phosphorylation can produce such byproducts as ROS and RNS. When excessive free radicals accumulate internally beyond an organism’s antioxidant capacity, oxidative stress can severely damage all cells (Ďuračková, 2014). Patients diagnosed with MDD have been found to show excessive peroxide and NO, with high levels of ROS and RNS (Maes et al., 2011). Early studies evaluated various oxidative and antioxidant indicators to investigate the mechanisms of oxidative stress-related diseases. For example, SOD, GSH, and MDA levels, as well as total T-AOC, have been measured (Bilici et al., 2001; Cumurcu et al., 2010; Sarandol et al., 2010). If overproduced, NO is a highly destructive free radical. However, NO at low concentrations is considered an important neurotransmitter related to the pathophysiology of neurological disorders. Compared with those in non-suicidal patients, the NO concentrations in depressed and suicidal patients are increased (Lu et al., 2014). Oxidative stress contributes to the pathophysiologic cascade related to hippocampal injury (Ahmad et al., 2010). Similarly, accumulating evidence indicates that oxidative stress is fundamental to CUMS-induced pathogenesis in rodents (Sakr et al., 2015; Li et al., 2016). Recently, the potential antioxidant and antidepressant effects of FGF-2 attracted interest (Rodrigues et al., 2014). Thus, we investigated whether FGF-2 protects the serum, prefrontal cortex, and hippocampus against CUMS-induced oxidative stress. In our study, CUMS mice exhibited significantly increased concentrations of MDA and NO and markedly reduced GSH levels, SOD and T-AOC activity in the serum, prefrontal cortex, and hippocampus. However, these parameters were significantly reversed by FGF-2 administration, suggesting that FGF-2 attenuates oxidative stress in CUMS mice.

Other neurotrophic factors, such as BDNF (Arancibia et al., 2008), have also highlighted the concerning role of neurogenesis in the pathogenesis of depression. Preclinical studies demonstrated that unforeseeable stress was related to decreased BDNF synthesis in the hippocampus and frontal cortex (Zhang et al., 2014) and that directly infusing BDNF into the hippocampus or midbrain induces antidepressant effects (Hoshaw et al., 2005). Other studies have shown that the chronic administration of antidepressants increases BDNF mRNA and protein expression in the hippocampus and cerebral cortex (Calabrese et al., 2011). Therefore, studying whether FGF-2 affects the expression and activity of BDNF as an indicator of depression would be interesting. In our study, BDNF protein expression was markedly reduced in the hippocampus and prefrontal cortex of depressed mice, while HMW FGF-2 significantly enhanced BDNF activity to a greater extent than did LMW FGF-2, suggesting potentially different effects of HMW and LMW FGF-2 in the CUMS model.

**FIGURE 7:**
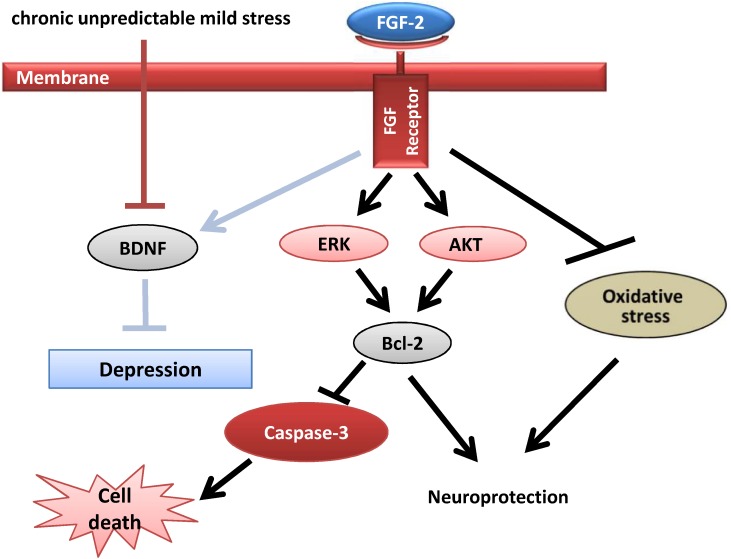
Potential mechanism of the antidepressant effect of FGF-2. FGF-2 activates the ERK and AKT signaling pathways by acting on FGFR1, which upregulates the expression of the antiapoptotic protein Bcl-2, leading to decreased caspase-3 levels. Upregulated BDNF protein expression ameliorates the symptoms of CUMS-induced depression, which may in turn increase Bcl-2 expression and inhibit caspase-3. In addition, the inhibition of oxidative stress exerts antidepressant effects on CUMS-induced mice.

Fibroblast growth factor 2 is known to activate two major signaling pathways, AKT and ERK, which contribute to organismal development and neuroprotection (Almeida et al., 2005). In addition, the ERK and AKT signaling pathways play a crucial role in the pathogenesis of many neurological diseases (Cheng et al., 2014). In our study, the ERK and AKT signaling pathways were activated by both HMW and LMW FGF-2 in the hippocampus and prefrontal cortex of CUMS mice. Furthermore, the decrease in Bcl-2 protein expression and increase in active caspase-3 levels caused by chronic stress were prevented by FGF-2 treatment. This discovery was consistent with Bcl-2 and caspase-3 being the downstream targets of AKT and ERK signaling, which were activated by FGF-2 in the hippocampus and prefrontal cortex (**Figure 7**). Our data further indicate that both FGF-2 isoforms also similarly increase AKT and ERK phosphorylation in the brain. The AKT and ERK signaling pathways were shown to be involved in the ability of HMW FGF-2 to ameliorate depressive symptoms in the CUMS model, which was consistent with a previous study showing that AKT and ERK were similarly activated in neurons by HMW and LMW FGF-2 (Cheng et al., 2016). The present report is the first to show that ERK and AKT pathway activation, Bcl-2 upregulation and caspase-3 inhibition are all required for HMW FGF-2 to improve depressive symptoms in mouse models of non-hereditary depression. Since our data did not show significant differences in the abilities of FGF-2 isoforms to mediate AKT and ERK activation and influence Bcl-2 and caspase-3 expression, the stronger antiapoptotic effect induced by HMW FGF-2 likely involves other signaling pathways. These findings further indicate that HMW FGF-2 functions similarly to LMW FGF-2 as a neurotrophic factor, exerting its antidepressant effect via receptor binding, which in turn activates the AKT and ERK signaling pathways. The antiapoptotic effects of FGF-2 appear to be similar to those of BDNF. Both similarly activate AKT and ERK signaling, increasing Bcl-2 expression and inhibiting caspase-3 activity to mediate neuroprotective effects (Guo et al., 2016; Phillips, 2017). Another possibility is that FGF-2 may indirectly exert an antiapoptotic effect by upregulating BDNF expression, which in turn increases Bcl-2 expression and inhibits caspase-3 activation.

## Conclusion

This study confirmed the antidepressant effects and underlying mechanisms of different FGF-2 isoforms in a CUMS model. Further investigation of different FGF-2 isoforms may reveal new research targets and therapeutic strategies for the prevention and treatment of depression.

## Author Contributions

YC and X-YQ conceived and designed the study. LW, X-XL and XC performed the experiments. LW and X-YQ did statistical analyses. YC drafted the manuscript. All authors critically revised the manuscript.

## Conflict of Interest Statement

The authors declare that the research was conducted in the absence of any commercial or financial relationships that could be construed as a potential conflict of interest.
