# Supplementary material for: Antidepressant-Like Effects of Low- and High-Molecular Weight FGF-2 on Chronic Unpredictable Mild Stress Mice
Source: Front Mol Neurosci. 2018 Oct 12;11:377. doi: 10.3389/fnmol.2018.00377 (PMC6194172; doi:10.3389/fnmol.2018.00377)
Supplement: Supplementary file 1 [file Data_Sheet_1.PDF]

A

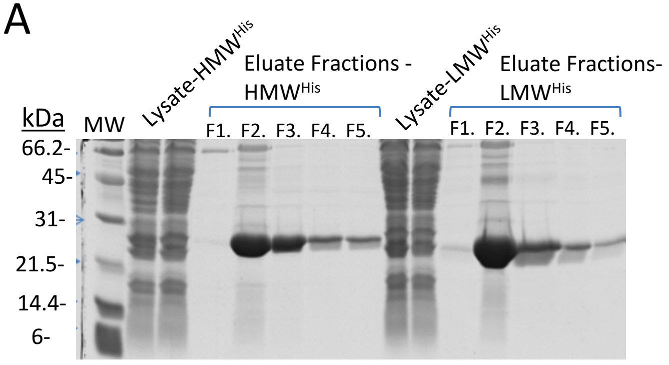

Total Lysates from E.Coli producing the FGF2 isoforms were fractionated in Nickel columns to produce purified His-tagged HMW- or LMW-FGF2, as indicated. Lysates and five purification column fractions are analyzed by SDS/PAGE and stained with Coomassie Blue

B

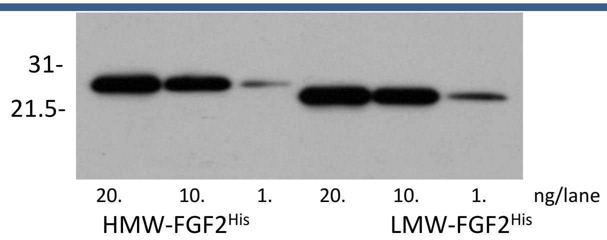

Western Blot for FGF2. Pooled fractions from the Hi- and Lo-FGF2 purifications were analyzed at 1, 10 and 20 ng/lane/each, as indicated, and processed for FGF2 detection by western blotting using monoclonal anti-FGF2 antibodies (UBI). The His-tag causes slower mobility to the FGF2 isoforms

C

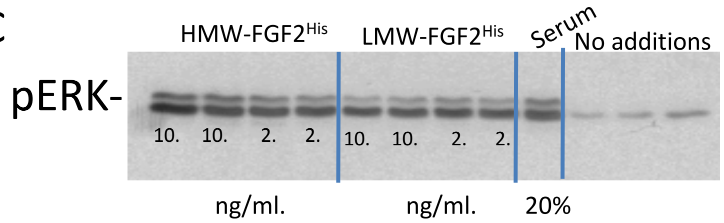

Testing the activity of FGF2 isoforms / ability to activate ERK. Primary rat heart fibroblasts in 0.5% fetal bovine serum were stimulated for 30 min with 20% serum, or 2-10 ng/ml of HMW- or LMW-FGF2, as indicated. Cell lysates (10 microg/lane) were analyzed for activation of ERK by probing with anti-phospho-ERK antibodies (pERK). Both types of isoforms were found to be capable of pERK upregulation compared to unstimulated cells.
